# Single‐Dose Pharmacokinetics of Intranasal Levetiracetam in Healthy Dogs

**DOI:** 10.1111/jvp.70046

**Published:** 2026-01-20

**Authors:** Jessica L. Wagner, Kari D. Foss, Jennifer M. Reinhart, Lauren E. Forsythe

**Affiliations:** ^1^ Department of Veterinary Clinical Medicine, College of Veterinary Medicine University of Illinois at Urbana‐Champaign Urbana Illinois USA; ^2^ University of Findlay College of Pharmacy Findlay Ohio USA

**Keywords:** cluster seizures, emergency management, epilepsy

## Abstract

Cluster seizures and status epilepticus in dogs are emergencies requiring rapid intervention. Intranasal (IN) benzodiazepines are effective for early seizure cessation, but the pharmacokinetics of longer‐acting antiseizure medications administered IN have not been investigated in dogs. This study aimed to describe the single‐dose pharmacokinetics of a compounded IN levetiracetam product (IN‐LEV) in healthy dogs. We hypothesized that the administration of IN‐LEV to healthy dogs will demonstrate similar pharmacokinetic parameters to IV administration. In a randomized crossover design, nine healthy dogs received a single 30 mg/kg IV dose (100 mg/mL) or a single 30 mg/kg IN dose (460 mg/mL) of levetiracetam. Serum levetiracetam concentrations were serially measured over 24 h. Pharmacokinetic analysis was performed using non‐compartmental methods and comparisons between routes of administration were made using the Wilcoxon signed‐rank test. *C*_max_, *T*_max_, and *t*_1/2_ for IN‐LEV were 14.6 ± 5.4 μg/mL, 2.3 ± 1.5 h, and 3.6 ± 0.4 h, respectively. IN‐LEV achieved minimum target concentrations (5 μg/mL) within 0.34 ± 0.22 h and maintained these levels for 6.57 ± 3.17 h. Bioavailability for IN‐LEV was 70% ± 27.4%. This study demonstrates that IN levetiracetam rapidly achieves the lowest reference interval concentration, but the high end of the interval was not achieved in any dog with a single 30 mg/kg dose. IN‐LEV may be a viable alternative for emergent seizure management when IV access is unavailable, but multiple doses may be required to achieve seizure cessation in some patients.

## 1 Introduction

An estimated 20%–40% of dogs with epilepsy do not have satisfactory seizure control, many of which may also experience cluster seizures (CS) or status epilepticus (SE) (Bray et al. 2021; Eagleson et al. 2012; Muñana et al. 2012; Peters et al. 2014). Mortality rates associated with SE and CS range from 35.3% to 38.3% (Bateman and Parent 1999; Bray et al. 2021; Hardy et al. 2012). SE and CS require rapid and effective delivery of antiseizure medications (ASMs) in order to prevent brain injury and other secondary systemic complications from prolonged seizure activity (Charalambous et al. 2017, 2019; Peters et al. 2014).

Intravenous (IV) administration of benzodiazepines is considered the treatment of choice for SE and CS in dogs. However, IV access may not be feasible if the patient is not within a hospital setting or if it cannot be quickly achieved. These situations demonstrate a need for developing other effective first‐line management of emergent seizures for use at home or in hospital environments when IV access is not available (Charalambous et al. 2024).

The intranasal (IN) route is an attractive method of emergency ASM administration for owners. Previous studies have utilized a mucosal atomization device (MAD) to administer benzodiazepines. The MAD is a syringe attachment that converts a liquid drug to a fine mist, which is distributed into the nasal cavity (Charalambous et al. 2017, 2019). When administered IN, benzodiazepines rapidly achieve target serum concentrations expected to control seizures (Bailey et al. 2017; Eagleson et al. 2012; Kapoor et al. 2016; Musulin et al. 2011; Peters et al. 2014; Platt et al. 2000; Shringarpure et al. 2021). While effective at early seizure cessation, benzodiazepines may require repeated dosing and have the potential for loss of efficacy over time (Cagnotti et al. 2019; Peters et al. 2014). Additionally, a multimodal pharmaceutical approach to cessation of SE and CS may be more beneficial than a single agent (Charalambous et al. 2024).

Levetiracetam (LEV) is now a commonly used ASM in dogs with epileptic seizures, and is commercially available in both oral and injectable formulations (Mastrocco et al. 2024). Levetiracetam (LEV) also has a high therapeutic index and excellent safety profile in dogs; its pharmacokinetic properties have been extensively investigated for intravenous, oral, intramuscular, and rectal administration (Beasley and Boothe 2015; Cagnotti et al. 2018; Dewey et al. 2008; Moore et al. 2010; Patterson et al. 2008; Peters et al. 2014). Intranasal administration of LEV in mice has rapid systemic absorption and similar pharmacokinetic properties as IV administration (Gonçalves et al. 2019). Additionally, LEV brain concentrations in mice were higher after IN administration compared to IV, which suggests that IN administration could provide direct nose‐to‐brain delivery of a longer‐acting ASM for use in CS or SE (Gonçalves et al. 2019).

The feasibility and pharmacokinetics of IN LEV have not been evaluated in dogs. The concentration of commercially available injectable LEV (100 mg/mL) would make IN administration difficult due to the volume of drug needed for typical IV doses. However, a highly concentrated LEV solution could be compounded to provide an appropriate dose and low volume for IN use. Therefore, the aim of this study was to establish the pharmacokinetic properties of such an IN‐LEV product in healthy dogs. We hypothesized that the administration of levetiracetam intranasally to healthy dogs would demonstrate pharmacokinetic parameters similar to intravenous administration, including the time at which levetiracetam concentration would first exceed 5 μg/mL and the duration of time that levetiracetam concentration would exceed 5 μg/mL. A therapeutic target of 5 μg/mL was selected based on the low end of the population therapeutic reference interval recommended in human seizure patients (Bazil 2002).

## 2 Materials and Methods

### 2.1 Study Population

Nine healthy dogs belonging to individuals associated with the University of Illinois College of Veterinary Medicine were enrolled. Dogs were eligible if they were at least 1 year of age and weighed a minimum of 15 kg. All dogs were determined to be in good health based on a complete physical examination and results of a complete blood count, serum biochemical profile, and urinalysis. To test tolerance of intranasal medication administration, all dogs were administered a test dose of intranasal saline at study admission. The volume of saline administered to each dog was equal to the volume of planned intranasal levetiracetam dose (30 mg/kg, equivalent to 0.065 mL/kg based on concentration of compounded LEV), delivered using a mucosal atomization device (MAD), and divided approximately by half into each nostril. Dogs were excluded if they had clinically relevant abnormal physical examination findings such as upper respiratory signs, abnormal laboratory results, or were receiving oral medications other than routine prophylactics. Additionally, any dog in which the tolerance test was not able to be performed was also excluded. Each owner provided informed consent and all procedures were approved by the University of Illinois Urbana‐Champaign Institutional Animal Care and Use Committee (protocol #22008).

### 2.2 Study Design

This was a two‐way, randomized, cross‐over study. Dogs were randomly assigned to receive either an IV dose (30 mg/kg) of a commercial 100 mg/mL levetiracetam (Hospira Inc., Lake Forest, IL) or an IN dose (30 mg/kg) of a compounded solution (460 mg/mL). Dogs underwent a washout period of no < 7 days and then received LEV by the opposite route. A minimum 7‐day washout period was elected to ensure complete elimination of levetiracetam, which has a plasma half‐life of 2–4 h (Dewey et al. 2008; Isoherranen et al. 2001; Moore et al. 2010; Patterson et al. 2008). Random assignment was performed using a randomization function in Microsoft Excel (Microsoft Corporation 2020).

Initial compounding and stability testing of the IN‐LEV formulation was performed from March 2022 to June 2022. Administration and sampling took place in January and February 2023.

### 2.3 IN Formulation

The use of commercially available LEV (100 mg/mL injectable) intranasally would require 1 mL per 3 kg of body weight to meet the desired dose of 30 mg/kg. This volume of drug delivered intranasally was unlikely to be tolerated. Therefore, an intranasal formulation was compounded to allow a dose of 30 mg/kg to be administered in a small volume.

Compounding was performed by a licensed pharmacist (LF) following the United States Pharmacopeia guidelines (Pharmacopeia 2024). The IN formulation of LEV was prepared by dissolving USP grade LEV bulk powder (Fagron, St. Paul, MN) in bacteriostatic saline (Hospira Inc., Lake Forest, IL) through continuous stirring to produce a solution with a final concentration of 460 mg/mL. This final concentration was selected with the goal to minimize the total volume administered intranasally. This was the maximum concentration that could be achieved without precipitation of the drug. Filtration was not performed. Visual inspection of the product was performed to ensure the compound remained in solution without precipitation. The stability of the compounded solution was evaluated in accordance with USP standards at a commercial laboratory (ARL Biopharma, Oklahoma City, OK) on Days 0, 30, 60, and 90 to establish a beyond‐use date (BUD). A single batch was prepared for stability testing through the commercial lab (ARL Biopharma). At each timepoint, 1 sample preparation was performed and analyzed, and the product was stored at 25°C and 60% relative humidity. The formulation was considered stable if the measured concentration was within ±10% of the target concentration at pre‐specified timepoints (USP 2024). One sample was analyzed at each timepoint. After completion of the stability testing, a new batch was prepared as described immediately prior to this IN‐LEV pharmacokinetic study. The final product for use in this study was stored in a plastic amber oral dosing vial (Clarke Container, Erie, PA) at room temperature in a cabinet protected from light until use.

### 2.4 Drug Administration and Sample Collection

All dogs were fasted at least 12 h prior to sedation for centrally inserted venous catheter (CVC) placement with 3–5 μg/kg dexmedetomidine (Dexdomitor; Orion Pharma; Espoo, Finland) and 0.4 mg/kg butorphanol (Torbugesic; Zoetis, Kalamazoo, MI, USA) intravenously. A single‐lumen, 18ga (4Fr)‐25 cm CVC (MILA International, Florence, KY, USA) was placed in the jugular vein of all dogs using the modified Seldinger technique (Beal and Hughes 2000). While under sedation, an 18ga or 20ga cephalic intravenous catheter (Sur‐Vet Surflo IV Catheter; Terumo Medical Corp., Somerset, NJ, USA) was also placed in dogs receiving IV‐LEV. Following completion of CVC placement, reversal of the dexmedetomidine was achieved with intramuscular administration of 0.05 mg/kg atipamezole (Antisedan; Orion Pharma; Espoo, Finland).

After at least 12 h following sedation, a baseline (time 0) blood sample was obtained and placed into a plastic, additive‐free red‐top tube (BD Vacutainer Serum Tubes) (4.0 mL) (Becton, Dickinson and Company, Franklin Lakes, NJ, USA). Dogs were then administered either IV‐LEV or IN‐LEV. IV‐LEV doses were administered via the peripheral catheter. For dogs receiving the IN dose, the compounded formulation was administered via a mucosal atomization device (MAD, Teleflex Medical, Morrisville, NC). The head was extended to elevate the nose, and the MAD was positioned into one nostril. Approximately half the volume was quickly administered into each nostril (over 1–2 s per nostril, with enough force to generate a fine mist from the MAD). Following administration, 3 mL blood samples were collected via the CVC into separate plastic red‐top tubes (BD Vacutainer Serum Tubes) (4.0 mL) (Becton, Dickinson and Company, Franklin Lakes, NJ, USA) at 0.25, 0.5, 0.75, 1, 2, 4, 8, 12, 18, and 24 h. Following coagulation and within two hours of collection, samples were centrifuged at room temperature for 10 min at 1900 × *g*. Serum was harvested and transferred to a polypropylene collection tube (Fisher Scientific, Hapton, NH, USA) and stored at −80°C until analysis.

All dogs were hospitalized from the time of sedation until the conclusion of sample collection. Heart rate (beats/min), respiratory rate (breaths/min), and body temperature (°F) were assessed prior to sedation and every 6 h during the sampling period, as well as a daily body weight. After complete recovery from sedation, all dogs had free access to water and were offered food every 12 h. All dogs were monitored for adverse drug effects (vomiting, diarrhea, ataxia, mentation/behavior changes) during the sampling period. All CVCs were removed prior to discharge.

### 2.5 Quantification of Serum Levetiracetam

Quantification of LEV in serum was performed using liquid chromatography‐mass spectrometry (LC–MS) (Carver Metabolomics Core Facility of the Roy J. Carver Biotechnology Center, University of Illinois at Urbana‐Champaign). A 10 μL aliquot of internal standard (levetiracetam‐d6, 10 ng/mL, Cayman Chemical, Ann Arbor, MI) was spiked at the beginning of extraction into samples. Chromatography was performed on a Vanquish LC system (Thermo Scientific, Waltham, MA, USA), with Hypersil GOLD, 2.1 × 150 mm (1.9 μ) column (Thermo Scientific, Waltham, MA, USA); the flow rate was 300 μL/min. The mobile phases were (A) 0.1% formic acid in water and (B) 0.1% formic acid in acetonitrile. The linear gradient was as follows: 0–0.5 min, 5% B; 0.5–3 min, 98% B; 3–4.5 min, 97% B; 4.6–6.5 min, 5% B. The injection volume was 1 μL. The column chamber temperature was 500°C. The mass spectrometer was a TSQ Altis LC–MS system (Thermo Scientific). Data were acquired in positive SRM mode at 1500 V with the following transitions: levetiracetam m/z 171.0 → m/z 126.0, levetiracetam‐d6 m/z 177.1 → m/z 132.1. Peak integration and quantitation using calibration curves adjusted for internal standard were performed with Thermo TraceFinder (4.1) software (Thermo Scientific). The calibrated concentration range was 2.5–2500 ng/mL. The low limit of quantitation was 2.5 ng/mL. All standard curves had a correlation coefficient value larger than 0.998 with a 1/*x* weighting factor. Dilutions were performed with blank dog serum as diluent. Recovery, accuracy, precision, and stability were within acceptable limits for an LC–MS assay (Tables S7–S9).

### 2.6 Pharmacokinetic and Statistical Analysis

Continuous data are presented as geometric mean ± standard deviation. Noncompartmental pharmacokinetic analysis of serum LEV‐IV and LEV‐IN administration were performed using commercially available software (Phoenix WinNonLin version 8.3, Certara USA Inc., Princeton, NJ). The absolute bioavailability of the intranasal levetiracetam was calculated by the AUC method: *F* = AUC_IN_/AUC_IV_. The time between administration and the first timepoint at which serum levetiracetam concentrations were > 5 μg/mL (*T*_>5_) and the longest duration during which serum levetiracetam concentrations were > 5 μg/mL (*T*_>5_dur_) are also reported. A minimum value of 5 μg/mL was selected as a target concentration based on the low end of the population therapeutic reference interval of 5–45 μg/mL recommended in humans for seizure control (Bazil 2002). Data were assessed for normality using the Kolmogorov–Smirnov test. *T*_>5_dur_ was compared between the formulations using a Wilcoxon signed‐rank test.

Data were also inspected for the number of dogs achieving serum levetiracetam concentrations above the middle (25 μg/mL) and high end (45 μg/mL) of the human population therapeutic reference interval. Additionally, multidose predictions were performed using the WinNonLin Nonparametric Superposition function. Serum levetiracetam concentrations were predicted following two consecutive doses (at 0 and 15 min) and three consecutive doses (0, 15, and 30 min) given in rapid succession. Predicted concentrations were compared against various possible target concentrations.

## 3 Results

### 3.1 Study Population

Nine dogs were included in this study (Table S1). These included 4 spayed females, 1 intact female, 3 neutered males, and 1 intact male. Mean age was 4.9 ± 2.4 years (range 1.6–9.9 years). Mean weight was 26.2 ± 6.8 kg (range 19.1 to 36.6 kg). The following breeds were represented: mixed breed dog (*n* = 3), Beagle (*n* = 1), American Pitbull Terrier (*n* = 1), Rhodesian ridgeback (*n* = 1), Catahoula Leopard Dog (*n* = 2), and Brittany Spaniel (*n* = 1). All dogs demonstrated unremarkable physical exams, laboratory profiles, and tolerated intranasal administration of saline.

### 3.2 IN LEV Stability and Antimicrobial Efficacy

Stability testing was performed following USP guidelines. The formulated 460 mg/mL IN‐LEV solution was found to maintain stability for the duration of the testing period at 30, 60, and 90‐day time points. Specifically, the formulation concentration was 100.3% of target concentration at 0 days, 100.6% at 30 days, 101.6% at 60 days, and 101.0% at 90 days. The formulation was considered stable if the measured concentration was within ±10% of the target concentration at pre‐specified timepoints (USP 2024). Aerobic microbial count and combined yeast/mold count were measured at 90 days. Both aerobic microbial and yeast/mold counts yielded < 10 CFU/mL and met the acceptance criteria for Nasal Route of Administration as defined by USP (Pharmacopeia 2024). There was a time lapse of 1 day between preparation and initial (Day 0) concentration testing due to overnight shipping. Otherwise, dates of stability testing reflected the age of the prepared solution. Based on stability testing for this product, a beyond use date (BUD) of 90 days is established. Following the results of stability testing, a new batch of IN‐LEV was prepared for immediate use in this study. The total time duration from preparation to the end of the study period was < 90 days; therefore, only a single batch of IN‐LEV was prepared for this study.

### 3.3 Pharmacokinetics

**FIGURE 1:**
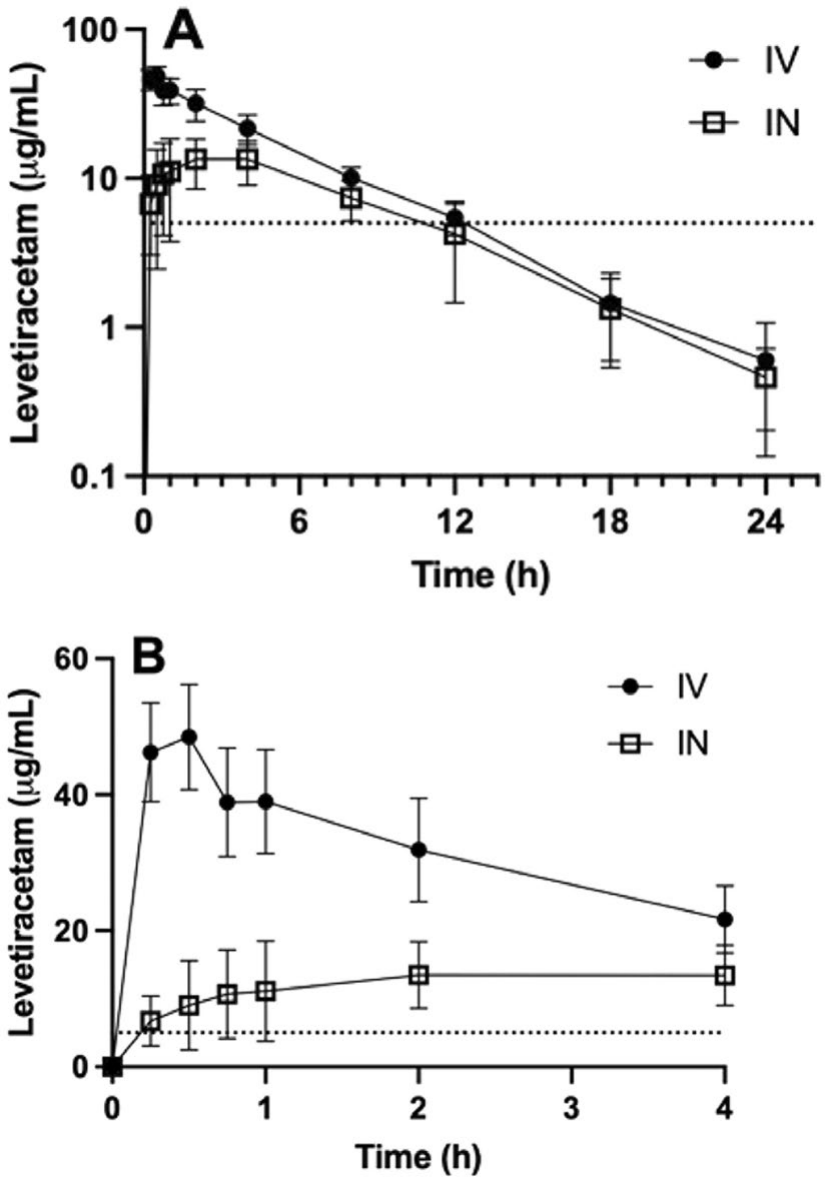
(A,B) Time‐Concentration curves following single‐dose administration of intravenous (IV) and intranasal (IN) levetiracetam.

**FIGURE 2:**
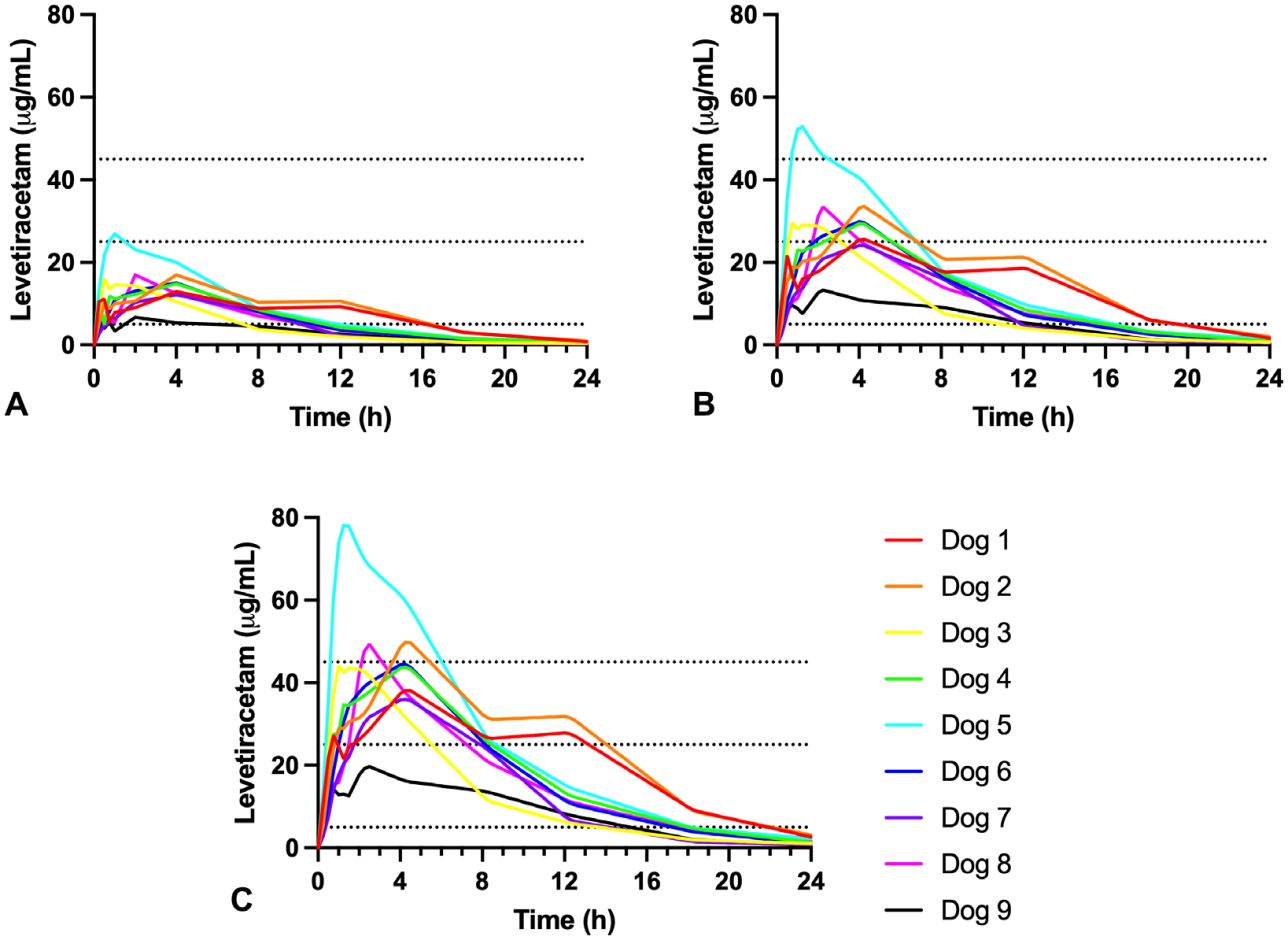
Time‐concentration curves for individual dogs following single IN dose (A), and predicted concentrations following two doses at 0 and 15 min (B), and three doses at 0, 15, and 30 min (C).

Pharmacokinetic parameters for single‐dose IV‐LEV and IN‐LEV are presented in Table 1. Time‐concentration curves are shown in Figure 1. LEV concentrations and pharmacokinetic parameters for individual dogs are presented in the Tables S2–S5. *T*_max_ for IN‐LEV was 2.3 ± 1.5 h (range 0.5–4 h). *C*_max_ for IN‐LEV was 14.6 ± 5.4 μg/mL (range 6.7–26.9 μg/mL). Calculated bioavailability (*F*) of IN‐LEV was 70% ± 27.4%. For IV‐LEV, concentrations were > 5 μg/mL at the first measured time point (*T*_>5_ = 0.25 ± 0.0 h) in all dogs, as expected for IV administration. For IN‐LEV, *T*_>5_ was 0.34 ± 0.22 h with 6/9 dogs reaching minimum target concentration range at 0.25 h, 1/9 at 0.5 h, and 2/9 at 0.75 h. IN‐LEV administration led to serum concentrations above the target (*T*_>5_dur_) for 6.57 ± 3.17 h (range 2.0–11.75 h), which was a statistically shorter duration than that achieved for IV administration (9.77 ± 2.11 h, range 7.75–11.75 h, *p* = 0.047). Only one dog achieved serum LEV concentrations above the middle of the target range (25 μg/mL) and no dogs achieved LEV concentrations above the high end of the target range (45 μg/mL) after a single IN dose (Figure 2A).

**TABLE 1:** Pharmacokinetic parameters for dogs administered intravenous (IV) and intranasal (IN) levetiracetam.

Parameters	Unit	Intravenous				Intranasal			
		Mean	SD	Min	Max	Mean	SD	Min	Max
*λ*_*z*_	h^−1^	0.178	0.041	0.128	0.267	0.192	0.023	0.162	0.225
*t*_1/2_	h	3.9	0.8	2.6	5.4	3.6	0.4	3.1	4.3
*C*_0_	μg/mL	47.8	8.9	33.3	61.1	—	—	—	—
*T*_max_	h	—	—	—	—	2.3	1.5	0.5	4.0
*C*_max_	μg/mL	—	—	—	—	14.6	5.4	6.7	26.9
AUC_0–*t*_	μg*h/mL	252.3	38.3	214.3	339.5	131.0	43.3	67.8	197.9
AUC_0–∞_	μg*h/mL	256.5	39.8	218.1	346.1	133.4	44.6	68.6	202.2
AUC_%Extrap_	%	1.2	1.2	0.2	4.2	1.7	0.6	1.1	2.5
*V*_*z*_	mL/kg	655.9	120.5	469.9	859.9	—	—	—	—
Cl	mL/h/kg	117.0	15.9	86.7	137.6	—	—	—	—
AUMC_0–*t*_	μg*h^2^/mL	1224.1	236.4	1063.1	1688.8	868.4	387.1	477.3	1602.5
AUMC_0‐∞_	μg*h^2^/mL	1340.1	334.0	1076.9	1976.1	938.0	423.4	499.9	1739.0
AUMC_%Extrap_	%	6.9	5.2	1.1	19.2	7.0	2.4	4.5	10.5
MRT	h	5.2	0.8	4.5	7.0	7.0	1.1	5.6	9.0
*V*_ss_	mL/kg	611.2	112.1	473.8	797.6	—	—	—	—
*F*	%	—	—	—	—	70.0	27.4	43.1	127.0
*T*_>5_	h	0.25	0.00	0.25	0.25	0.34	0.22	0.25	0.75
*T*_>5_dur_	h	9.77	2.11	7.75	11.75	6.57	3.17	2.00	11.75

*Note:* Geometric mean, standard deviation (SD), minimum value (Min), and maximum value (Max) are presented.

Abbreviations: *λ*_*z*_, terminal rate constant; *C*_0_, predicted concentration at time 0 h; *T*_max_, time at which maximum concentration is achieved; *C*_max_, maximum concentration; AUC_0–*t*_, observed area under the curve; AUC_0–∞_, area under the curve extrapolated to infinity; AUC_%Extrap_, % AUC extrapolated; *V*_*z*_, volume of distribution by the area method; Cl, clearance; AUMC_0–*t*_, observed area under the moment curve; AUMC_0–∞_, area under the moment curve extrapolated to infinity; AUMC_%Extrap_, % AUMC extrapolated; MRT, mean residence time; *V*_ss_, volume of distribution at steady state; *F*, absolute bioavailability; *T*_>5_, time at which levetiracetam concentration first exceeded 5 μg/mL; *T*_>5_dur_, longest duration for which levetiracetam concentration exceeded 5 μg/mL; *t*_1/2_, terminal half‐life.

### 3.4 Multidose Predictions

After two 30 mg/kg IN doses at 0 and 15 min (Figure 2B), 7/9 dogs were predicted to reach or exceed concentrations of 25 μg/mL (middle of the target range) with one dog predicted to exceed the upper end of the target range (> 45 μg/mL). Following three IN doses at 0, 15, and 30 min (Figure 2C), 3/9 dogs had predicted concentrations that exceeded the upper limit of the target range, and only 1/9 dog's predicted concentrations did not reach the middle of the target range.

### 3.5 Adverse Events

The only immediate adverse effect observed was sneezing, which occurred in all dogs after receiving IN‐LEV. Sneezing was mild and self‐limiting and did not require medical intervention. One dog (Dog 5) experienced mild nasal congestion for 1 day following discharge after receiving IN‐LEV, which resolved without additional therapy. After receiving IV‐LEV, the same dog (Dog 5) experienced mild irritation on the ventral neck 2 days following routine CVC removal. This was treated with topical antiseptic for 1 week and resolved. One dog (Dog 2) experienced 2 episodes of vomiting approximately 18–20 h after receiving IN‐LEV. Following collection of the final 24 h sample, this dog was monitored and treated with maropitant (1 mg/kg SC). The underlying cause for vomiting in this patient was undetermined but the vomiting did not continue following discharge. No additional adverse events were observed in this population of dogs.

## 4 Discussion

The aim of this study was to describe the pharmacokinetic behavior of a compounded formulation of LEV administered intranasally, while also assessing the feasibility of this route of administration of LEV in dogs. Results of this single‐dose pharmacokinetic study demonstrate that LEV is absorbed following IN administration and maintains minimum target serum concentrations for several hours. Thus, IN‐LEV may have a role in the acute management of CS and SE in dogs. This study also demonstrates the feasibility of compounding a stable intranasal solution and delivery in dogs, which can support additional investigations into repeat dosing pharmacokinetics and/or efficacy studies.

The ability to administer emergency anti‐seizure medications at home is beneficial when early intervention is key to successful treatment (Charalambous et al. 2021). The IN route is attractive due to the limitations with other routes for ASMs. Alternative routes of ASMs have been investigated in dogs including oral (PO), rectal, and intranasal (IN) administration. Administering ASMs by mouth can be easily performed by clients at home but success may be hindered by postictal impairment of swallowing ability and increased risk of aspiration pneumonia. Initiation of oral treatments may be delayed by the duration of the seizure or post‐ictal period, as well as slower drug absorption by oral administration (Cagnotti et al. 2020). Rectal administration of ASMs (including benzodiazepines, zonisamide, and levetiracetam) has also been described, but may be viewed as inconvenient. Medications delivered rectally can have variable and unpredictable absorption, depending on the drug used (Brewer et al. 2015; Cagnotti et al. 2018; Musulin et al. 2011; Papich and Alcorn 1995; Peters et al. 2014; Schwartz et al. 2013).

IN administration is used commonly in human medicine for a wide variety of drugs in emergency settings (Bailey et al. 2017). The respiratory and olfactory regions have high vascularization and permeability, making them the primary sites targeted for drug absorption (Charalambous et al. 2017). This indirect pathway from nose to brain involves rapid drug absorption by the large, highly vascular nasal epithelium and delivery to the brain via systemic circulation. A direct pathway is also possible via the trigeminal nerve (ophthalmic and maxillary branches that innervate the respiratory mucosal epithelium) (Charalambous et al. 2021; Kapoor et al. 2016). IN pathway for drug delivery provides many advantages including ease of administration and direct drug delivery to the brain. This is of particular interest concerning first‐line ASMs offering rapid and effective treatment of seizures (Charalambous et al. 2017, 2019).

In the current study, the *C*_max_ for IN‐LEV was 14.6 ± 5.4 μg/mL, which is generally lower than (Patterson et al. 2008). *C*_max_ reported for levetiracetam administered by other non‐intravenous routes to dogs. Peters et al. demonstrated a *C*_max_ of 36 μg/mL following rectal administration (Peters et al. 2014). However, that study used a higher dose of levetiracetam (40 mg/kg), which might explain the higher concentrations observed. Additionally, they did not include an IV phase for bioavailability calculation, so it is possible that rectal levetiracetam is better absorbed than IN, also increasing *C*_max_. Patterson et al. demonstrated a *C*_max_ of 30.3 μg/mL after IM administration. This study used a lower levetiracetam dosage (20 mg/kg) than ours but found higher bioavailability (113%) for IM administration. Also, the *T*_max_ for IM administration (0.67 h) was shorter than what we found for IN administration (2.3 h). Thus, it appears that both the rate and extent of absorption explain the differences in *C*_max_ observed between these two routes. In previous studies of PO LEV administration, *C*_max_ ranged from 21.9 to 59.9 μg/mL; however, variable doses (16–27 mg/kg) and formulations (immediate‐ vs. extended‐release) make comparison between the IN and PO routes difficult (Beasley and Boothe 2015; Boozer et al. 2015; Moore et al. 2010, 2011; Muñana et al. 2012; Patterson et al. 2008).

The *T*_max_ of IN LEV was 2.3 ± 1.5 h, which is longer compared to other routes of administration. In one study that compared IV, IM, and PO LEV in dogs, *T*_max_ of IM administration was 0.67 h, and PO administration was 1.35 h (Patterson et al. 2008). Rectal LEV had a *T*_max_ of 1.7 h (Peters et al. 2014). The longer *T*_max_ with IN‐LEV may reflect prolonged absorption from the nasal cavity compared to other routes of administration. Despite this, 6/9 dogs in this study reached minimum target concentrations by 0.25 h following IN administration, which was the first post‐administration timepoint measured. It is possible that some dogs in this study could have reached LEV concentrations within the target reference interval before 15 min, but these earlier time points were not measured. The remaining 3 dogs reached target concentrations by 30 or 45 min. It is unclear why a delay in target concentrations occurred; however, 2 of these 3 dogs (dogs 7 & 9) had the lowest *C*_max_ and *F* in the study. Assuming linear absorption by the IN route, it would take these dogs longer to reach a minimum serum concentration of 5 μg/mL, so the delay could be explained by poor bioavailability or loss of product. In some dogs, it is possible that some of the volume administered reached the nasopharynx and was subsequently swallowed. We tried to avoid this by use of the atomization device, but this does not guarantee that inadvertent oral administration didn't occur. The pharmacokinetics of the oral route of administration for this compounded LEV product has not been studied. Inadvertent partial PO administration could have affected *T*_max_ and other parameters, but the degree and significance of this effect are not known.

The AUC_0–*t*_ was lower for IN versus IV administration (131.0 ± 43.3 vs. 252.3 ± 38.0 μg*h/mL) resulting in a bioavailability (*F*) of 70% ± 27.4%. This difference may be due to true reduced absorption across the nasal mucosa. Alternately, it might have resulted from inadvertent dose reduction due to sneezing and/or runoff of product. The volume administered for IN LEV ranged from 1.2 to 2.4 mL per dog. While this is a significant reduction from the volume that would be required if administered using the commercially available 100 mg/mL formulation (5.5–22 mL), some loss of drug was expected. If LEV could have been formulated to a concentration > 460 mg/mL, allowing an even smaller volume to be administered, there might have been less drug loss and higher IN‐LEV bioavailability; however, this was precluded by solubility of the compound. A bioavailability of 70% for IN administration of LEV is lower than what has been reported for intramuscular (IM), PO immediate‐release, and PO extended‐release LEV, which is near 100% in dogs (Beasley and Boothe 2015; Patterson et al. 2008). However, pharmacokinetic studies for IN benzodiazepines revealed bioavailability for IN diazepam was 41%, and IN midazolam ranged from 52% to 74% (Eagleson et al. 2012; Musulin et al. 2011). Therefore, bioavailability of IN LEV is similar to other IN ASMs studied in dogs and achieves concentrations within the human reference interval.

Terminal half‐life was statistically shorter for IN‐LEV (3.6 ± 0.4 h vs. 3.9 ± 0.8 h). The route of administration should not affect the terminal half‐life, except in the case of flip‐flop kinetics, which are not demonstrated in this pharmacokinetic study. Therefore, this statistical finding is likely spurious (type 2 error). The duration that LEV concentrations remained above 5 μg/mL (*T*_>5_dur_) was 6.57 ± 3.17 h. This was statistically shorter compared to IV‐LEV (9.7 ± 2.11 h), but the clinical value of this finding is unclear. Our selected minimum target concentration of 5 μg/mL was extrapolated from the concentrations expected in humans receiving therapeutic doses of levetiracetam (5–45 μg/mL) (Bazil 2002). However, other human references suggest different ranges and effective LEV plasma concentrations have not been established in dogs (Couderc et al. 2022). Regardless of the specific lower and upper bounds used, a population reference therapeutic interval acknowledges that different patients respond to LEV at different minimum concentrations and target concentrations must be individualized during therapeutic drug monitoring. To address this inherent interindividual variation, we evaluated LEV concentrations following IN administration against the middle and upper ends of the target range (25 and 45 μg/mL). However, no dog achieved sustained concentrations above these more stringent targets. Therefore, we also assessed multidose predictions for IN‐LEV for 2–3 doses at 15 min intervals. Multidose predictions suggest that serum concentrations are likely to exceed the middle of the target range and may exceed the upper limit of the target range in some dogs. These calculations suggest that multiple IN‐LEV doses may be beneficial in achieving higher serum concentrations (and potentially more effective seizure control) in emergency seizure scenarios. This 15–30 min timeline was chosen as a reasonable interval to evaluate for clinical response to treatment and could allow for continued emergency intervention at home or en route to a veterinary hospital.

Early intervention in the case of SE or CS is critical to a successful outcome (Charalambous et al. 2017). Therefore, the benefits of ease of at‐home administration outweigh a potentially shortened duration of action in this scenario, particularly for LEV, which is a longer‐acting ASM compared to benzodiazepines (Musulin et al. 2011; Schwartz et al. 2013). In fact, combination of an IN benzodiazepine with IN‐LEV could provide the best emergency therapy for SE or CS at home, with the benzodiazepine providing rapid seizure cessation and LEV extending the duration of seizure control until veterinary care can be accessed. Such a protocol would also be beneficial for dogs with SE or CS that are developing resistance to benzodiazepines.

This study is not without limitations. First, a compounded product for IN‐LEV was formulated in‐hospital for the purpose of this study. The decision to compound an intranasal solution from bulk drug was multifactorial. First, a highly concentrated product was desired to reduce the volume needed to achieve the same dose that was administered IV and to improve tolerability. To the authors' knowledge, there are no published recommendations regarding intranasal drug volume in veterinary patients. However, publications in human medicine suggest that low volume of administration improves absorption and therapeutic effects while minimizing discomfort associated with nasal deposition (Gao et al. 2020). The product used in this study was compounded from bulk substance, which is justified in cases where the desired product cannot be prepared from commercially available products. A solution was desired to increase the chance of sufficient absorption. This could not be achieved by manipulating commercially available oral tablets due to the added excipients in these products.

The use of a compounded solution may contribute to differences in absorption. The stability of compounded formulations is not always known and may depend on how the medication is prepared. Compounded drugs do not undergo consistent testing for identity, quality, strength, purity, and stability; therefore, the results of research described in reports using compounded products may not be reproducible. Stability testing of this product supports a beyond use date (BUD) of 90 days, which may be used as long as the formulation, packaging, and storage remain the same. At each timepoint, only a single sample was analyzed for stability testing; ideally, multiple replicates would be analyzed to confirm these results. The batch of IN‐LEV submitted for stability testing was not the same batch prepared for use in this pharmacokinetic study. However, the second batch was prepared, packaged, and stored in the same manner following USP guidelines, and would be subject to the 90‐day BUD established by the previous stability testing. In the absence of stability testing, this product would have had a default BUD of 35 days, as it was prepared with bacteriostatic saline and is classified as an aqueous preserved nonsterile compound (Pharmacopeia 2024). In practice, this product would not be available for office stock due to restrictions in the FDA Guidelines for Industry #256 and current approved bulk substances list. Our IN‐LEV product was not filtered prior to storage and use in this study. While there was no evidence of precipitation of drug noted at any time during stability testing or pharmacokinetic study, this does not rule out the possibility of microparticles present in solution. Undissolved particles in the solution would not be absorbed and therefore negatively impact bioavailability (Behl et al. 1998).

This population of dogs included only medium to large‐breed dogs with mesocephalic skull conformation. It is unknown if dogs of variable facial conformation would absorb IN‐LEV similarly. All IN doses were delivered to alert, non‐sedated patients. A consistent immediate effect of IN LEV was sneezing or nasal runoff. This did not occur in the pre‐study tolerability testing, which may indicate that this drug and concentration may be more irritating to the nasal mucosa than normal saline alone. Sneezing and nasal runoff likely resulted in unavoidable and unmeasurable loss of the product, and therefore lower peak drug concentrations. It is unknown how much drug would be lost in an animal in an ictal or post‐ictal state. It has been shown that added agents that improve mucosal adhesion may improve contact time and absorption (by reducing runoff). For example, one study in dogs showed that IN administration of a hydroxypropyl methylcellulose midazolam gel preparation had superior performance in peak plasma concentration and bioavailability compared to IN and rectal administration of commercially available midazolam solution (Eagleson et al. 2012). Finally, dilutional parallelism was not included in the LEV LC‐MS assay validation so reported concentrations above the upper end of the working range of the assay may be less accurate than those within the working range.

Multidose predictions were performed after analyzing single‐dose data. While higher serum concentrations in a similar timeframe are possible with multiple doses, future studies should be performed to confirm these predictions. This study did not evaluate CSF LEV concentrations, and therefore cannot determine the potential nose‐to‐brain benefit of IN‐LEV delivery, which has been demonstrated in mice (Gonçalves et al. 2019). Future investigation in CSF LEV concentrations in dogs following IN versus IV administration would be of interest to compare serum and CSF concentrations. If high CSF concentrations can be achieved with IN administration, it is possible a smaller dose and lower volume of drug would be required for IN administration, which may improve tolerability and absorption.

## 5 Conclusion

This study demonstrated that IN administration of LEV reaches the minimum concentrations of the target reference interval (5 μg/mL) rapidly and is able to maintain concentrations within this interval for several hours. However, the low end of this reference interval may not be a sufficient serum concentration to control seizures in all dogs, and multiple doses may be required to achieve higher concentrations, which may be more effective for controlling seizures in some patients. IN‐LEV may be a feasible option for emergency treatment of CS or SE. Additional studies are warranted to evaluate the efficacy of this route of administration in the treatment of CS and SE. A randomized clinical trial comparing IV and IN‐LEV in the cessation of seizure activity could yield useful findings to help guide in‐hospital and out‐of‐hospital recommendations for dogs suffering from epilepsy who experience CS and SE.

## Author Contributions

J.L.W. contributed to the study planning, acquisition of samples, and first draft of the manuscript. K.D.F. contributed to study conception and design and obtained funding for the study. J.M.R. contributed to study conception and design, acquisition of data, pharmacokinetic analysis, and interpretation of data. L.E.F. contributed to study conception and design, and formulation of the compounded drug. All authors contributed to writing and revisions of the manuscript.

## Funding

This work was supported by the University of Illinois Companion Animal Research Grant Program ‐ Wayne D. and Josephine H. Spangler Fund.

## Ethics Statement

The authors confirm that the ethical policies of the journal, as noted on the journal's author guidelines page, have been adhered to and the appropriate ethical review committee approval has been received. The authors confirm that they have adhered to either US or European standards for the protection of animals used for scientific purposes.

## Conflicts of Interest

The authors declare no conflicts of interest.

## Supporting information


**Data S1:** jvp70046‐sup‐0001‐supinfo.docx.

## Data Availability

The data that supports the findings of this study are available in the Supporting Information of this article.
