# Supplementary material for: Single‐Dose Pharmacokinetics of Intranasal Levetiracetam in Healthy Dogs
Source: J Vet Pharmacol Ther. 2026 Jan 20;49(2):110–9. doi: 10.1111/jvp.70046 (PMC12968516; doi:10.1111/jvp.70046)

Supplemental Data

**Table 1. Demographic Data for Individual Dogs**

|  | Age (years) | Sex | Breed | Weight (kg) |
| --- | --- | --- | --- | --- |
| Dog 1 | 3.8 | FS | American pit bull terrier | 19.7 |
| Dog 2 | 6 | FS | MBD | 36.6 |
| Dog 3 | 4.3 | FS | Beagle | 19.1 |
| Dog 4 | 1.6 | MC | MBD | 35.8 |
| Dog 5 | 3.3 | FS | Rhodesian Ridgeback | 32.4 |
| Dog 6 | 5.8 | MC | MBD | 22.8 |
| Dog 7 | 6.1 | MI | Catahoula Leopard Dog | 25.5 |
| Dog 8 | 9.9 | FI | Catahoula Leopard Dog | 22.1 |
| Dog 9 | 3.6 | MC | Brittany Spaniel | 22.6 |

FS = female, spayed; MC = male, castrated; MI = male, intact; FI = female, intact; MBD = mixed breed dog

RAW DATA TABLES

**Table 2. Serum concentrations for individual dogs administered intravenous levetiracetam.**

| **Time (h)** | **Levetiracetam (ug/mL)** | | | | | | | | |
| --- | --- | --- | --- | --- | --- | --- | --- | --- | --- |
|  | **Dog 1** | **Dog 2** | **Dog 3** | **Dog 4** | **Dog 5** | **Dog 6** | **Dog 7** | **Dog 8** | **Dog 9** |
| 0 | 0.00 | 0.00 | 0.00 | 0.00 | 0.00 | 0.00 | 0.00 | 0.00 | 0.00 |
| 0.25 | 49.95 | 39.70 | 33.33 | 55.89 | 40.40 | 51.51 | 44.25 | 51.40 | 49.62 |
| 0.5 | 60.05 | 39.32 | 51.35 | 56.08 | 37.65 | 43.43 | 54.81 | 48.78 | 44.63 |
| 0.75 | 36.27 | 43.37 | 32.72 | 51.99 | 23.97 | 36.11 | 42.97 | 38.40 | 44.07 |
| 1 | 27.32 | 40.86 | 36.65 | 50.12 | 27.42 | 39.57 | 46.44 | 41.68 | 40.93 |
| 2 | 27.10 | 32.86 | 21.11 | 41.14 | 28.61 | 46.03 | 30.08 | 32.72 | 27.06 |
| 4 | 22.69 | 18.88 | 16.23 | 32.26 | 18.18 | 26.51 | 18.43 | 21.62 | 20.34 |
| 8 | 9.32 | 11.33 | 11.02 | 13.63 | 8.97 | 8.88 | 7.77 | 10.00 | 10.11 |
| 12 | 4.59 | 7.30 | 6.22 | 5.88 | 5.41 | 4.43 | 7.12 | 3.36 | 4.69 |
| 18 | 1.13 | 2.96 | 0.78 | 2.47 | 1.87 | 1.43 | 0.21 | 1.26 | 0.94 |
| 24 | 0.52 | 1.52 | 0.00 | 0.98 | 0.86 | 0.61 | 0.11 | 0.41 | 0.41 |

**Table 3. Serum concentrations for individual dogs administered intranasal levetiracetam.**

| **Time (h)** | **Levetiracetam (ug/mL)** | | | | | | | | |
| --- | --- | --- | --- | --- | --- | --- | --- | --- | --- |
|  | **Dog 1** | **Dog 2** | **Dog 3** | **Dog 4** | **Dog 5** | **Dog 6** | **Dog 7** | **Dog 8** | **Dog 9** |
| 0 | 0.05 | 0.01 | 0.00 | 0.00 | 0.05 | 0.00 | 0.00 | 0.00 | 0.00 |
| 0.25 | 10.52 | 5.22 | 7.61 | 10.78 | 13.54 | 4.32 | 2.79 | 5.02 | 4.65 |
| 0.5 | 11.11 | 10.09 | 16.08 | 4.85 | 21.71 | 6.17 | 4.65 | 4.51 | 4.10 |
| 0.75 | 5.58 | 8.72 | 13.49 | 11.81 | 25.23 | 8.07 | 6.68 | 5.85 | 5.44 |
| 1 | 7.82 | 10.04 | 14.56 | 11.25 | 26.91 | 10.95 | 6.34 | 5.52 | 3.36 |
| 2 | 9.05 | 10.64 | 14.37 | 12.29 | 23.13 | 13.11 | 10.35 | 16.99 | 6.73 |
| 4 | 12.95 | 16.98 | 10.52 | 14.83 | 20.06 | 15.03 | 12.16 | 12.44 | 5.38 |
| 8 | 8.81 | 10.36 | 3.78 | 8.53 | 8.75 | 8.13 | 7.96 | 6.94 | 4.53 |
| 12 | 9.30 | 10.63 | 1.99 | 4.21 | 4.87 | 3.56 | 2.21 | 3.73 | 2.65 |
| 18 | 3.05 | 2.96 | 0.65 | 1.56 | 1.61 | 1.29 | 0.49 | 1.39 | 0.67 |
| 24 | 0.78 | 0.95 | 0.34 | 0.40 | 0.70 | 0.33 | 0.25 | 0.53 | 0.18 |

**Table 4. Pharmacokinetic parameters for individual dogs administered intravenous levetiracetam.**

| **Parameter** | **Unit** | **Dog 1** | **Dog 2** | **Dog 3** | **Dog 4** | **Dog 5** | **Dog 6** | **Dog 7** | **Dog 8** | **Dog 9** |
| --- | --- | --- | --- | --- | --- | --- | --- | --- | --- | --- |
| λ_z_ | h^-1^ | 0.189 | 0.128 | 0.204 | 0.149 | 0.153 | 0.267 | 0.169 | 0.175 | 0.205 |
| t_1/2_ | h | 3.7 | 5.4 | 3.4 | 4.7 | 4.5 | 2.6 | 4.1 | 4.0 | 3.4 |
| C_0_ | μg/mL | 50.0 | 40.1 | 33.3 | 55.9 | 43.3 | 44.3 | 61.1 | 54.2 | 55.2 |
| AUC_0-t_ | μg*h/mL | 237.1 | 271.2 | 214.3 | 339.5 | 222.2 | 238.8 | 281.8 | 247.0 | 239.4 |
| AUC_0-∞_ | μg*h/mL | 239.9 | 283.1 | 218.1 | 346.1 | 227.9 | 239.2 | 285.4 | 249.3 | 241.4 |
| AUC_%Extrap_ | % | 1.1 | 4.2 | 1.7 | 1.9 | 2.5 | 0.2 | 1.3 | 0.9 | 0.8 |
| V_z_ | mL/kg | 660.2 | 829.8 | 673.8 | 581.9 | 859.9 | 469.9 | 620.9 | 687.8 | 606.3 |
| Cl | mL/h/kg | 125.1 | 106.0 | 137.6 | 86.7 | 131.7 | 125.4 | 105.1 | 120.3 | 124.3 |
| AUMC_0-t_ | μg*h^2^/mL | 1120.7 | 1595.9 | 1063.1 | 1688.8 | 1208.5 | 1065.1 | 1245.0 | 1087.3 | 1103.0 |
| AUMC_0-∞_ | μg*h^2^/mL | 1200.4 | 1976.1 | 1150.0 | 1891.8 | 1380.6 | 1076.9 | 1352.8 | 1157.1 | 1160.2 |
| AUMC_%Extrap_ | % | 6.6 | 19.2 | 7.6 | 10.7 | 12.5 | 1.1 | 8.0 | 6.0 | 4.9 |
| MRT | h | 5.0 | 7.0 | 5.3 | 5.5 | 6.1 | 4.5 | 4.7 | 4.6 | 4.8 |
| V_ss_ | mL/kg | 625.9 | 739.4 | 725.6 | 473.8 | 797.6 | 564.6 | 498.3 | 558.4 | 597.5 |
| T_>5_ | h | 0.25 | 0.25 | 0.25 | 0.25 | 0.25 | 0.25 | 0.25 | 0.25 | 0.25 |
| T_>5_dur_ | h | 7.75 | 11.75 | 11.75 | 11.75 | 11.75 | 7.75 | 11.75 | 7.75 | 7.75 |

λ_z_ = terminal rate constant; t_1/2_ = terminal half-life; C_0_ = predicted concentration at time 0 h; AUC_0-t_ = observed area under the curve; AUC_0-∞_ = area under the curve extrapolated to infinity; AUC_%Extrap_ = % AUC extrapolated; V_z_ = volume of distribution by the area method; Cl = clearance; AUMC_0-t_ = observed area under the moment curve; AUM =C_0-∞_ = area under the moment curve extrapolated to infinity; AUMC_%Extrap_ = % AUMC extrapolated; MRT = mean residence time; V_ss_ = volume of distribution at steady state; T_>5_ = time at which levetiracetam concentration first exceeded 5 μg/mL; T_>5_dur_ = longest duration for which levetiracetam concentration exceeded 5 μg/mL.

**Table 5. Pharmacokinetic parameters for individual dogs administered intranasal levetiracetam.**

| **Parameter** | **Unit** | **Dog 1** | **Dog 2** | **Dog 3** | **Dog 4** | **Dog 5** | **Dog 6** | **Dog 7** | **Dog 8** | **Dog 9** |
| --- | --- | --- | --- | --- | --- | --- | --- | --- | --- | --- |
| λ_z_ | h^-1^ | 0.207 | 0.201 | 0.172 | 0.190 | 0.164 | 0.197 | 0.218 | 0.162 | 0.225 |
| t_1/2_ | h | 3.4 | 3.4 | 4.0 | 3.7 | 4.2 | 3.5 | 3.2 | 4.3 | 3.1 |
| T_max_ | h | 4 | 4 | 0.5 | 4 | 1 | 4 | 4 | 2 | 2 |
| C_max_ | μg/mL | 12.9 | 17.0 | 16.1 | 14.8 | 26.9 | 15.0 | 12.2 | 17.0 | 6.7 |
| AUC_0-t_ | μg*h/mL | 166.5 | 194.4 | 101.5 | 142.6 | 197.9 | 135.3 | 106.1 | 126.4 | 67.8 |
| AUC_0-∞_ | μg*h/mL | 170.2 | 199.1 | 103.4 | 144.7 | 202.2 | 137.0 | 107.2 | 129.7 | 68.6 |
| AUC_%Extrap_ | % | 2.2 | 2.4 | 1.9 | 1.4 | 2.1 | 1.2 | 1.1 | 2.5 | 1.2 |
| AUMC_0-t_ | μg*h^2^/mL | 1415.5 | 1602.5 | 518.1 | 948.2 | 1132.0 | 866.0 | 641.5 | 839.5 | 477.3 |
| AUMC_0-∞_ | μg*h^2^/mL | 1524.4 | 1739.0 | 576.5 | 1009.3 | 1260.4 | 914.5 | 673.7 | 938.3 | 499.9 |
| AUMC_%Extrap_ | % | 7.1 | 7.8 | 10.1 | 6.1 | 10.2 | 5.3 | 4.8 | 10.5 | 4.5 |
| MRT | h | 9.0 | 8.7 | 5.6 | 7.0 | 6.2 | 6.7 | 6.3 | 7.2 | 7.3 |
| F | % | 127.0 | 88.0 | 50.1 | 53.4 | 91.3 | 84.9 | 49.8 | 81.1 | 43.1 |
| T_>5_ | h | 0.25 | 0.25 | 0.25 | 0.25 | 0.25 | 0.50 | 0.75 | 0.25 | 0.75 |
| T_>5_dur_ | h | 11.75 | 11.75 | 3.75 | 7.25 | 7.75 | 7.50 | 7.25 | 7.25 | 2.00 |

λ_z_ = terminal rate constant; t_1/2_ = terminal half-life; T_max_ = time at which maximum concentration is achieved; C_max_ = maximum concentration; AUC_0-t_ = observed area under the curve; AUC_0-∞_ = area under the curve extrapolated to infinity; AUC_%Extrap_ = % AUC extrapolated; AUMC_0-t_ = observed area under the moment curve; AUM =C_0-∞_ = area under the moment curve extrapolated to infinity; AUMC_%Extrap_ = % AUMC extrapolated; MRT = mean residence time; F = absolute bioavailability; T_>5_ = time at which levetiracetam concentration first exceeded 5 μg/mL; T_>5_dur_ = longest duration for which levetiracetam concentration exceeded 5 μg/mL.

**ASSAY VALIDATION**

**LC-MS Methods Description**

Serum samples were analyzed for levetiracetam using liquid chromatography-mass spectrometry (LC-MS) by the Carver Metabolomics Core Facility of the Roy J. Carver Biotechnology Center, University of Illinois Urbana-Champaign.

A 10 uL aliquot of internal standard (levetiracetam-d6, 10 ng/mL, Cayman Chemical, Ann Arbor, MI) was spiked at beginning of extraction into samples. Chromatography was performed on a Vanquish LC system (Thermo Scientific, Waltham, MA, USA), with Hypersil GOLD, 2.1 x 150 mm (1.9μ) column (Thermo Scientific, Waltham, MA, USA); the flow rate was 300 μL/min. The mobile phases were (A) 0.1% formic acid in water and (B) 0.1% formic acid in acetonitrile. The linear gradient was as follows: 0-0.5 min, 5% B; 0.5-3 min, 98% B; 3-4.5 min, 97% B; 4.6-6.5 min, 5% B. The injection volume was 1 μL. The column chamber temperature was 500°C. The mass spectrometer was a TSQ Altis LC-MS system (Thermo Scientific). Data were acquired in positive SRM mode at 1500V with the following transitions: levetiracetam m/z 171.0 🡪 m/z 126.0, levetiracetam-d6 m/z 177.1 🡪 m/z 132.1. Peak integration and quantitation using calibration curves adjusted for internal standard were performed with Thermo TraceFinder (4.1) software (Thermo Scientific). The linear range of the assay was 2.5 – 2,500 ng/mL. The intra- and interassay coefficients of variation were 6.7-15.8% and 2-12%, respectively, for concentrations relevant to the study.

**Table 6. Mass spectrometer information**

| Compound | Precursor (m/z) Q1 | Product (m/z) Q3 | Collision Energy (V) | RF Lens (V) | Data |
| --- | --- | --- | --- | --- | --- |
| Levetiracetam | 171.0 | 68.9 | 29.11 | 30 | qualitative |
| Levetiracetam | 171.0 | 98.1 | 25.13 | 30 | qualitative |
| Levetiracetam | 171.0 | 126.0 | 16.07 | 30 | quantitative |
| Levetiracetam | 171.0 | 154.0 | 10.23 | 30 | qualitative |
| Levetiracetam d6 | 177.1 | 74.0 | 29.79 | 32.59 | qualitative |
| Levetiracetam d6 | 177.1 | 104.1 | 26.34 | 32.59 | qualitative |
| Levetiracetam d6 | 177.1 | 132.1 | 16.75 | 32.59 | quantitative |
| Levetiracetam d6 | 177.1 | 160.1 | 10.23 | 32.59 | qualitative |

Note: Quantitative data listed in the Table were the SRM used to determine concentrations in the samples. Qualitative data are generated from the qualifier ions from which we collect data to provide further confirmation of a positive identification.

**Method Validation**

1. Range and Linearity

The calibrated concentration range is 2.5 ng/mL – 2,500 ng/mL. The lowest quantitation limit is 2.5 ng/mL. Overall, the standard curves had a correlation coefficient value larger than 0.998 (the weighting factor is 1/x).

1. Table 7. Recovery

|  | **Levetiracetam spiked into serum (ng/mL)** | **Calculated concentration of Levetiracetam (ng/mL)*** | **Recovery % †** |
| --- | --- | --- | --- |
|  | 10 | 10.3 ± 1.8 | 105.7% ± 4.7% |
| **Recovery (n=5)** | 100 | 105.2 ± 6.7 | 99.9% ± 6.1% |
|  | 2500 | 2434.0 ± 152.7 | 98.7% ± 8.0% |

* The listed values are mean calculated concentration of levetiracetam in samples used in the recovery evaluation.

**†** Recovery is calculated based on % of internal standard peak area in serum samples / internal standard peak area in 50% methanol.

1. Table 8. Accuracy and precision

|  | **Within-run accuracy (n=5)** | | | **Between-run accuracy** | **Within-run precision** | **Between-run precision** |
| --- | --- | --- | --- | --- | --- | --- |
|  |  | **(Mean ± SD)** |  | **n=15** | **n=15** | **n=15** |
| **(ng/mL)** | **Day 1** | **Day 2** | **Day 3** |  |  |  |
| 10 | 113.6±21.7 | 119.7±15.7 | 93.7±14.2 | 109.1±3.6 | 15.8% | 12% |
| 100 | 93.4±12.4 | 96.0±6.9 | 101.3±12.2 | 96.6±1.8 | 10.8% | 4% |
| 2500 | 99. 7±7.4 | 96.2±5.0 | 97.3±7.2 | 97.7±1.8 | 6.7% | 2% |

Note: Each of the 3 days, we made new calibration curves in new serum.

1. Table 9. Stability

| (n=5) | **After 72 hours at 15°C *** | |  |
| --- | --- | --- | --- |
| **(ng/mL)** | **Mean ± SD** | **RSD (%) †** | **Concentration Change (%)** |
| 10 | 9.3 ± 1.8 | 19.0 | -14.5 |
| 100 | 76.3 ± 5.2 | 6.8 | -21.3 |
| 2500 | 2576 ± 162.9 | 6.3 | -5.5 |

Five replicates were performed at each concentration, each prepared separately.

* Sustaining the autosampler at 10**°**C for 3 days leads to condensation. Setting it at 15°C does not have this effect. Samples that are in our autosampler are typically sustained at 10°C but for only 1 day maximum. Thus, this 72 hour test at 15°C does not represent the typical scenario for samples being held in our autosampler for a sustained period.

**†** RSD (%) = Relative standard deviation percentage (also known as coefficient of variation – CV) of the samples tested for each of the concentration levels.

**LEVETIRACETAM STABILITY**


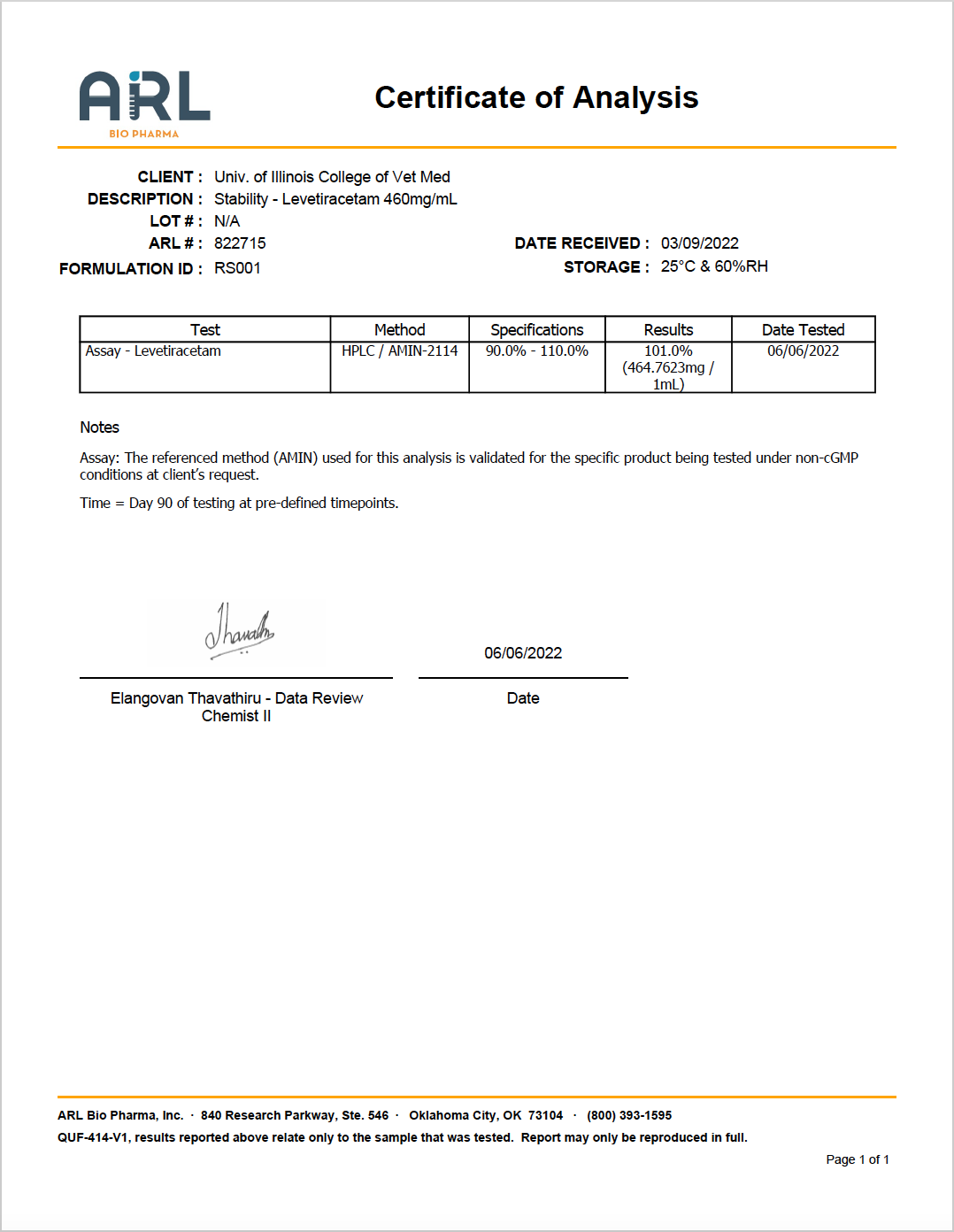


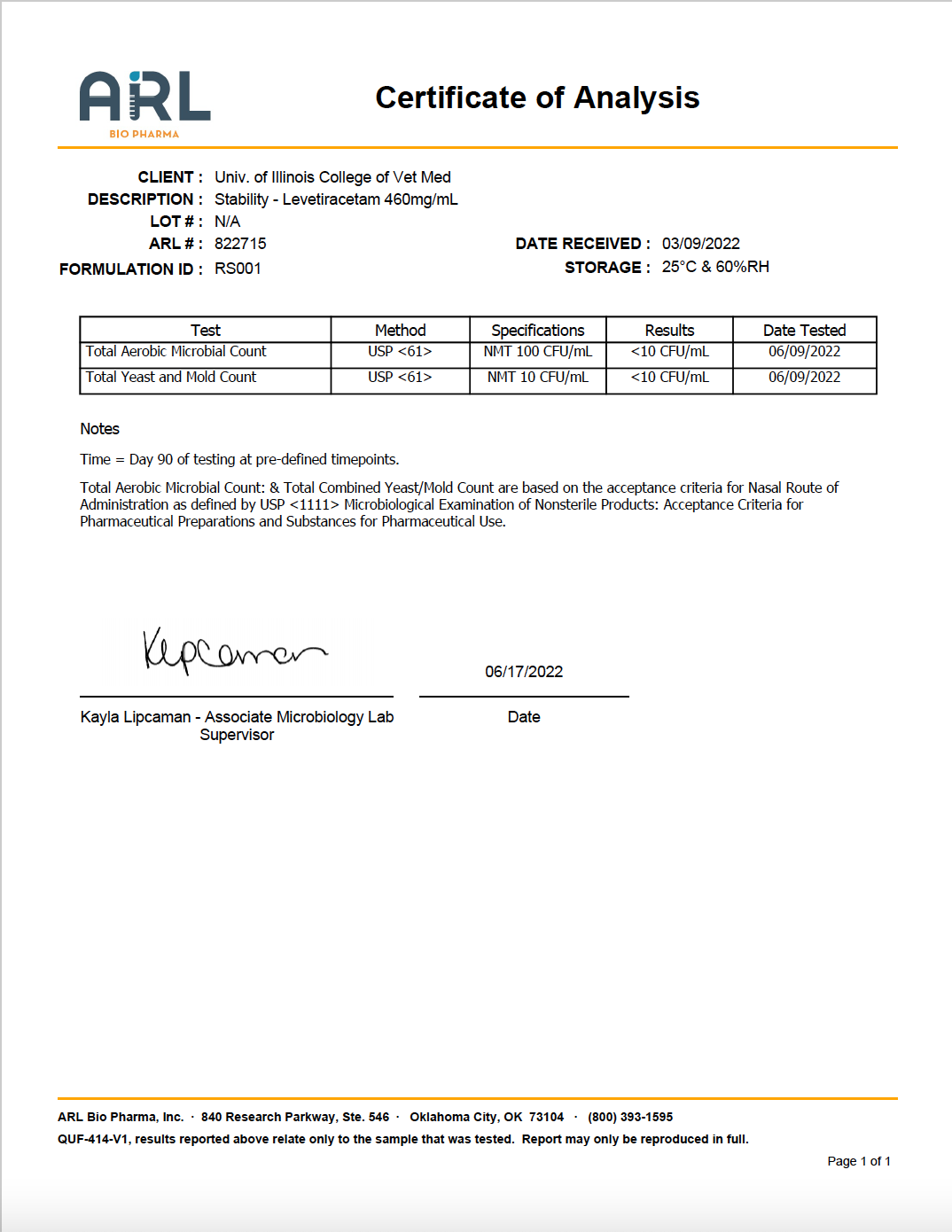

Supplement: Supplementary file 1 — Data S1: jvp70046‐sup‐0001‐supinfo.docx. [file JVP-49-110-s001.docx]
